# 

**Published:** 1811-11

**Authors:** 


### MONTHLY CATALOGUE OF MEDICAL BOOKS.

Anatomico-Chirurgical Views of the Male and Female Pelvis; with appropriate explanations and references to the Parts. By John James Watt, Surgeon. The engravings executed from original drawings, by Lewis. Folio. Coloured plates. Lond.

Practical Observations on Cancer. By the late John Howard, Fellow of the Royal College of Surgeons, and Surgeon Extraordinary to the Cancer ward in the Middlesex Hospital. 8vo. Lond.

Engravings of the Arteries; illustrating the second volume of the Anatomy of the Human Body, and serving as an Introduction to the Surgery of the Arteries. By Charles Bell. Third edition, with additional plates. Royal 8vo. Lond.

The Principles of Midwifery; including the Diseases of Women and Children. Second Edition, much enlarged. By John Burns, Lecturer on Midwifery, and Member of the Faculty of Physicians, and Surgeon of Glasgow. 8vo. Lond.

The Æsculapian Monitor; or, Faithful Guide to the History of the the Human Species, and most important branches of Medical Philosophy. By the Rev. Edward Barry, M. D. 8vo. Lond.

Vaccination Vindicated; or, an address to the People of England, upon the important subject of Vaccine Inoculation, wlth remarks on the necessity, in its behalf, of Legislative and Clerical interference. Written with a view to remove some prejudices inimical to its Progress, and to guide the Public to a right consideration of its great and real merits. 8vo. Norwich.

An Essay on the Disease called Yellow Fever, with observations concerning Febrile Contagion, Typhus Fever, Dysentery, and Plague, &c. &c. &c. By Edward Nathaniel Bancroft, M. D. 8vo. Lond.

### NOTICES TO CORRESPONDENTS.

Communications have been received from Messrs. R. Harrup, T. Smith, H. Davies, T. Mackell, A. Pearson, Surgeon to the English Factory, Canton, through Mr. Weeding; Medicus, &c. &c.

---

*Printed by E. Hemsted, Great New Street, Fetter Lane.*

